# The Influence of Cardiovascular Medications on Iron Metabolism in Patients with Heart Failure

**DOI:** 10.3390/medicina55070329

**Published:** 2019-07-02

**Authors:** Oana Sirbu, Victorita Sorodoc, Irina M. Jaba, Mariana Floria, Alexandra Stoica, Lenuta Profire, Cristina Tuchilus, Gabriela Rusu, Laurentiu Sorodoc

**Affiliations:** 1 Department of Internal Medicine, Gr. T. Popa University of Medicine and Pharmacy, 700115 Iasi, Romania; 2 Independent Researcher, 700115 Iasi, Romania; 3 Department of Pharmaceutical Chemistry, Gr. T. Popa University of Medicine and Pharmacy, 700115 Iasi, Romania; 4 Department of Microbiology, Gr. T. Popa University of Medicine and Pharmacy, 700115 Iasi, Romania; 5 Department of Pharmacology, Gr. T. Popa University of Medicine and Pharmacy, 700115 Iasi, Romania

**Keywords:** iron deficiency, heart failure, beta blockers, calcium-channel blockers, angiotensin-converting enzyme inhibitors

## Abstract

*Background and objectives:* The etiology of anemia associated with heart failure is not fully understood, but there are data suggesting the involvement of multiple mechanisms, including various drug therapies used in patients with heart failure. Our primary objective was to evaluate the impact of beta blockers, angiotensin-converting enzyme inhibitors, and calcium-channel blockers on iron metabolism in patients with heart failure. *Materials and Methods:* This was a prospective observational study that included patients diagnosed with heart failure and iron deficiency (defined by ferritin <100 μg/L, or 100–300 μg/L with transferrin saturation <20%). Patients with anemia secondary to a known cause were excluded. *Results:* We found a statistically significant correlation between beta-blocker treatment and ferritin values (*p* = 0.02). Iron, hemoglobin, and hematocrit levels were significantly lower in the patients using calcium-channel blockers than those who were not. We also found a statistically significant indirect correlation (*p* = 0.04) between the use of angiotensin-converting enzyme inhibitors and hematocrit levels. *Conclusion:* The contribution of our study arises from the additional data regarding the drug-induced etiology of iron deficiency. Practitioners should be aware of the potential impact of therapeutic recommendations and this should imply a close monitoring of the biochemical parameters of iron deficiency in this category of patients.

## 1. Introduction

Heart failure remains a major cause of mortality around the world, despite recent improvements in treatment. Anemia in patients with heart failure has a high incidence and is associated with less favorable outcomes [1]. Patients who suffer from anemia showed a higher rate of mortality, more frequent hospitalization, lower physical exercise tolerance and lower quality of life [2]. Furthermore, iron deficiency is associated with worse prognosis, independently of other well-established outcome predictors, including anemia [3]. The etiology of anemia associated with heart failure is not fully understood, but current data suggest the involvement of multiple mechanisms: iron deficiency [4], inflammation [5], low erythropoietin levels [6], hemodilution [7], and bone marrow dysfunction [8].

Several studies suggest the involvement of various pharmacological agents, such as beta-blockers (BBs) [9] and renin–angiotensin–aldosterone system blockers [10,11], in the etiology of anemia. Nevertheless, the conclusions regarding the impact of these therapies on the etiology of anemia stem from studies conducted for other purposes [9,10,11]. More than that, there are data suggesting that calcium-channel blockers (CCBs) may also have an impact on iron status [12].

These studies did not bring definitive conclusions and may have raised additional questions regarding the influence that different drug classes have on the iron status of patients with heart failure. Due to the impact of anemia on mortality and morbidity, it is vital to better understand the different etiologies involved in order to prevent the development of iron deficiency and screen more frequently for this condition in patients at risk.

In accordance with the latest guideline, diltiazem and verapamil are not recommended in heart failure with reduced ejection fraction because of their negative inotropic action and risk of worsening the disease. Amlodipine is recommended to reduce blood pressure when hypertension persists despite treatment with a combination of drugs indicated for heart failure and may be considered in patients unable to tolerate a BB to relieve angina. No direct indication is presented in heart failure with preserved ejection fraction (EF) without co-morbid disease [13].

In this context, an observational prospective study was conducted to evaluate the impact of BBs, angiotensin-converting enzyme inhibitors (ACEIs) and CCBs (amlodipine) on iron metabolism in patients with heart failure.

## 2. Materials and Methods

**Figure 1:**
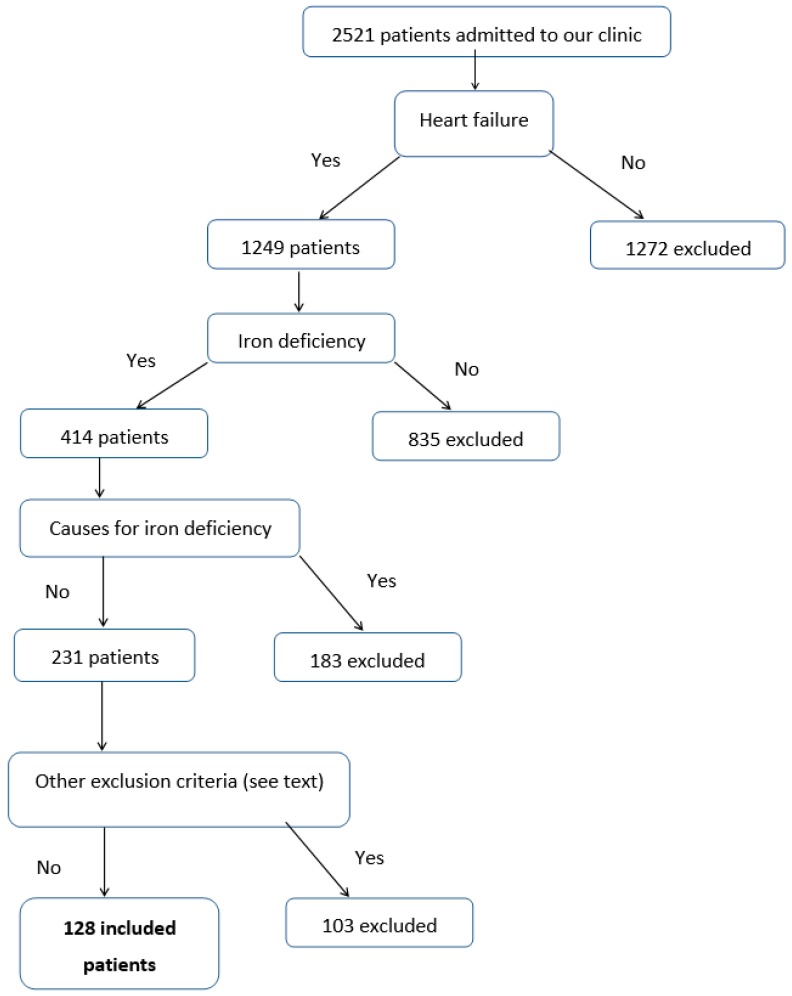
The algorithm for the selection of cases.

During a period of 12 months, 2501 patients consecutively admitted to the 2nd Internal Medicine Clinic of the “Sf. Spiridon” County Emergency Hospital from Iasi were clinically, biologically (NT-proBNP), and echocardiographically screened for the detection of heart failure (with preserved or reduced ejection fraction) (Figure 1). The criteria used for the diagnosis of heart failure were the following:

- Heart failure with preserved and mid-range EF: signs and symptoms of heart failure, a value of NT-proBNP > 125 pg/mL, a history of conditions that may lead to heart failure and the following echocardiographic changes: left ventricular hypertrophy (left ventricular wall >11 mm), left atrium enlargement or diastolic dysfunction (E/A < 1) and an EF > 40% [13].
- Heart failure with reduced EF: signs and symptoms of heart failure, a value of NT-proBNP >125 pg/mL, a history of conditions that could lead to heart failure and an EF < 40% [13].

In patients thus diagnosed with heart failure, blood tests were subsequently performed to confirm or exclude iron deficiency (iron, ferritin and transferrin saturation). Patients showing absolute iron deficiency (defined as ferritin <100 μg/L) or functional iron deficiency (defined by ferritin 100–300 μg/L with transferrin saturation <20%) [14] were assessed afterwards for other etiologies of iron deficiency. Those with the following pre-existing conditions or newly diagnosed diseases that may cause anemia were excluded: positive fecal occult blood test; neoplastic or inflammatory diseases, chronic liver disease; chronic blood loss due to gastrointestinal bleeding, recurrent epistaxis, hematuria, meno-/metrorrhagia; recent major surgery; severe anemia requiring transfusion; iron treatment within the previous 4 weeks; history of erythropoietin treatment; stage IV and V chronic kidney disease; or pregnancy. In addition, patients who underwent prescription changes within the past three months were also excluded from the study. The etiology of heart failure was represented by hypertension and ischemic heart disease.

After applying these criteria, a total of 128 patients were eligible for inclusion in the study.

A detailed past medical history and specific information about the BB, CCB and ACEI used within the previous three months were obtained and all patients underwent a thorough physical examination. Blood samples for complete blood count; iron, ferritin, and NT-proBNP levels; transferrin saturation; C-reactive protein (CRP); and creatinine were collected early in the morning after a minimum of eight hours of fasting. Iron, ferritin and transferrin were assayed with an Architect i device (Abbott Laboratories, Chicago, IL, USA) using Abbot reagents. NT-pro BNP levels were assayed with a Pathfast device (Mitsubishi, Tokyo, Japan) using Pathfast reagents.

All subjects gave their informed consent for inclusion before they participated in the study. The study was conducted in accordance with the Declaration of Helsinki, and the protocol was approved by the Ethics Committee of the “Gr. T Popa” University of Medicine and Pharmacy from Iasi (1.12.2015) and the Ethics Committee of the “Sf. Spiridon” County Emergency Hospital from Iasi (No 67/3.11.2016).

### Statistical Analysis

Continuous variables were presented as the mean and standard deviation (SD) or median and interquartiles 25th–75th (IQR 25–75). Groups were subsequently compared using the Mann–Whitney U test for two independent samples or Kruskal–Wallis test for multiple samples followed by pairwise comparisons. Categorical variables were expressed in frequencies and percentages. A z-test for column proportions followed by Bonferroni corrections for p-values were used to determine significant differences between frequencies in subgroups.

Following the transformation into dichotomous variables, point-biserial correlations were used to study correlations where one variable was categorical [15]. Cramer’s V coefficient was used to ascertain associations between categorical variables.

Interactions between beta blocker, CCB or ACEI treatment and New York Heart Association (NYHA) classification subgroups when influencing continuous variables were evaluated using General Linear Model (GLM) Univariate Analysis of Variance.

Data analysis was performed using IBM SPSS Statistics 20 for Windows (Version 20.0, IBM Corp., Armonk, NY, USA). All tests were two-tailed and a *p*-value <0.05 was considered statistically significant.

## 3. Results

Out of the 128 study patients, 54.68% were female and 45.31% were male, with an average age of 72.95 (range 38–98) years old. Based on the New York Heart Association (NYHA) classification, 46.09% were categorized as class II, 50.78% as class III and only 3.12% of the patients were categorized as NYHA class IV. In accordance with EF classifications, 53.54% of the patients were included in heart failure with reduced EF and 46.46% in heart failure with preserved EF.

Regarding the etiology of heart failure, 44.09% of the patients had ischemic heart disease and 88.97% had hypertension. BB treatment was used by 67.96% of the patients, ACEI by 62.5%, while 28.12% used CCB (amlodipine).

### 3.1. Beta Blockers and Iron Deficiency

No significant differences in age, hemoglobin, hematocrit, iron levels, or echocardiographic parameters were found between patients who followed a BB regimen and those who did not (Table 1). Ferritin levels were significantly higher in patients on BBs (*p* = 0.02). Patients in this category also had significantly higher NT-pro BNP levels (*p* = 0.024) (Table 1).

The patients on BB treatment showed proportions that did not differ significantly at the 0.05 level (z-test for column proportions) for either of the heart failure class subgroups.

To determine whether the changes observed in anemia parameter levels and NT-proBNP were predominantly due to BB treatment, or whether a more severe NYHA class is actually the cause and the use of BBs is simply more likely to be associated with disease severity, the interactions between BB and NYHA class were further investigated.

NYHA class scores did not differ significantly (*p* = 0.143) among treatment cohorts regardless of BB use (Table 1). No significant association between BB treatment and disease severity, illustrated by NYHA classification subgroups, were found (Cramer’s V 0.157, *p* = 0.37).

A General Linear Model Univariate Analysis of Variance also determined that there is no significant interaction between BB and NYHA class (*p* = 0.401) in influencing NT-proBNP levels. Likewise, no significant interaction between BB and NYHA class was found to influence ferritin (*p* = 0.976), hemoglobin (*p* = 0.114) or iron (*p* = 0.441) levels.

Furthermore, no significant associations were found between BB and EF subgroups (Cramer’s V 0.213, *p* = 0.092).

General Linear Model two-way ANOVA found no significant interaction between BB and EF categories in influencing NT-proBNP levels (*p* = 0.387). Moreover, no significant interaction was found between BB and EF categories in influencing ferritin (*p* = 0.763), hemoglobin (*p* = 0.77) or iron (*p* = 0.191) levels.

CRP levels showed no significant differences among the group of patients on BB versus those not receiving this treatment (*p* = 0.425) (Table 1).

No definite correlation was found between BB in general or specific BB and ferritin levels (BBr = 0.023, *p* = 0.8, carvedilol r = 0.024, *p* = 0.79; bisoprolol r = 0.155, *p* = 0.083; metoprolol r = 0.04, *p* = 0.653 and nebivolol r = −0.32, *p* = 0.722) (Table 2).

### 3.2. Amlodipine and Iron Deficiency

The characteristics of patients treated with amlodipine are shown in Table 3. Of the 36 patients using amlodipine, 24 (66.66%) were using BB at the same time. Iron (*p* = 0.038), hematocrit (*p* = 0.003) and hemoglobin (*p* = 0.018) levels were found to be significantly lower for the group of patients using CCBs compared to those who did not (Table 3). Similarly, NT-proBNP levels were significantly lower (*p* = 0.008) in these patients.

Comparing NYHA class subgroups, the proportions of patients using amlodipine do not differ significantly at the 0.05 level (z-test for column proportions) regardless of the severity of heart failure.

NYHA class scores did not differ significantly (*p* = 0.157) among treatment cohorts regardless of amlodipine use (Table 3). Additionally, no significant associations were found between CCB and NYHA classification subgroups (Cramer’s V 0.177, *p* = 0.26).

No significant interaction was found between amlodipine and heart failure class in influencingNT-proBNP (*p* = 0.143) levels. Moreover, no significant interaction was found between amlodipine and NYHA classification in influencing ferritin (*p* = 0.702), hemoglobin (*p* = 0.264) or iron (*p* = 0.383) levels.

Also, no significant associations were found between CCB and EF subgroups (Cramer’s V 0.115, *p* = 0.5).

A General Linear Model two-way ANOVA found no significant interaction between amlodipine and EF categories in influencing NT-proBNP levels (*p* = 0.28). Moreover, no significant interaction was found between amlodipine and EF categories in influencing ferritin (*p* = 0.127), hemoglobin(*p* = 0.87) or iron (*p* = 0.159) levels.

A moderate, statistically significant, indirect correlation was identified between amlodipine treatment and hematocrit (*p* = 0.008) or iron levels (*p* = 0.012) (Table 2).

### 3.3. ACEI and Iron Deficiency

Regarding patients treated with ACEI, no significant changes were found in hemoglobin (*p* = 0.097), hematocrit (*p* = 0.063), iron (*p* = 0.589) and ferritin (*p* = 0.333) levels when compared with patients not using this treatment (Table 4).

A significant, weak and indirect correlation (*p* = 0.04) was found between the ACEI treatment and hematocrit levels (Table 2).

## 4. Discussion

The study results show that BB and amlodipine could influence the iron status in patients with heart failure.

NT-pro BNP levels were significantly higher in patients with BB and amlodipine treatment, so this study investigated whether the relationship between treatment with these two classes of drugs and anemia parameters was influenced by the severity of the disease. The severity of the disease was expressed by NYHA class or EF subgroups.

No significant association was found between a specific treatment and NYHA class or EF categories. More specifically, the proportions of patients undergoing either BB or amlodipine were not found to be dependent on the NYHA class. Furthermore, no interactions were found between specific therapies and NYHA class/EF categories in controlling the biological parameters for iron deficiency anemia such as ferritin, iron and hemoglobin. These results are in alignment with observations from other authors, which pointed to the fact that NYHA class and EF do not necessarily stratify the prognosis in HF patients. [16]

All this supports the hypothesis that the variations in hemoglobin, iron or ferritin levels are influenced in a significant measure by the specific therapies, and not only by the severity of the underlying heart failure.

###  4.1. Impact of Beta Blockers on Iron Metabolism

Our study indicated an association between BB treatment and higher ferritin levels (*p* = 0.028) when compared to those following no treatment, while no significant changes were found in hemoglobin, hematocrit or iron levels.

Ferritin is an acute phase reactant and may be elevated in conditions causing inflammation or tissue injury [17]. In this context, CRP levels, another inflammatory marker, were evaluated in patients treated with BB, compared to those following another regimen. The results suggest that our findings are unlikely to have been influenced by the presence of inflammation.The COMET (Carvedilol Or Metoprolol European Trial) study results show that treatment with carvedilol (but not with metoprolol) induced a small but significant decrease in hemoglobin levels (*p* = 0.0047). This decrease was not associated with a negative therapeutic outcome. To the contrary, this study described a reduction in mortality for this category of patients. According to the authors, this result may have been due to the blocking action of carvedilol on β–1, β–2 and α adrenergic receptors. Specifically, the blockade of β–2 adrenergic receptors may lead to a decrease in erythropoietin production, followed by erythroid progenitor proliferation due to a sympathetic mechanism [9].

Other studies have shown that treatments with BB in general [18], or with carvedilol in particular [19], lead to a significant increase in hemoglobin levels after one year of treatment. BB treatment also entails an anti-inflammatory effect [20]. It is generally accepted that inflammation leads to increased hepcidin levels, which in turn causes a decrease in iron absorption at the intestinal level [5]. Because of the anti-inflammatory activity of BB [20], hepcidin levels may therefore decrease, leading to an increase in iron absorption at the intestinal level, probably leading to increased ferritin levels, a mechanism that may be involved in determining the results of our study. However, such a hypothesis will require subsequent studies of causality.

### 4.2. Amlodipine and Iron Status

There is insufficient data available regarding the effect of CCB on iron status. To our best knowledge, this is the first study evaluating the effect of CCB treatment on anemia and iron status in patients with heart failure. One of the reasons is that CCBs are not standard treatments for patients with heart failure.

In this study, amlodipine was found to be linked with lower hemoglobin (*p* = 0.018), hematocrit (*p* = 0.003) and iron (*p* = 0.038) levels. An indirect correlation between amlodipine use and hematocrit (r = −0.23, *p* = 0.008) and iron (r = −0.22, *p* = 0.012) levels was also determined. As all of our patients were using a dihydropyridine CCB (amlodipine), the results may be narrowed down to this specific class.

These findings are consistent with data from a study describing a decrease in hemoglobin and hematocrit levels in patients treated with CCB for arterial hypertension who also had chronic kidney disease (but no heart failure). In the group of patients treated with CCBs, the level of erythropoietin (EPO) was significantly higher compared with the patients not using CCBs [12].

Calcium ions are of key importance in the differentiation and proliferation of erythroid precursors. The stimulation of erythroid precursors by erythropoietin leads to higher levels of intracellular free calcium and adenosine monophosphate as well as the activation of protein kinases, followed by cell differentiation and proliferation [21]. It is crucial for EPO to cause an increase in free calcium levels in erythroid precursor cells in order to exhibit its proliferative activity. Low hemoglobin levels stimulate a rise in plasma EPO levels. In the pathophysiology of heart failure, EPO levels are elevated in response to global tissue hypoxia, but this elevation is not proportional to the detected hemoglobin levels, a mismatch caused by the resistance of hematopoietic bone marrow to EPO [22]. The mechanism behind this resistance is not yet fully understood, but treatment with CCBs may be involved to a significant degree. Larger trials are therefore needed to define the specific mechanism by which CCBs seem to be involved in lowering hemoglobin and hematocrit levels.

A recent meta-analysis [23] has shown a small association between CCB treatment and risk of gastrointestinal bleeding. However, these observations provide a likely explanation for the decreased iron levels in our study. Notwithstanding the exclusion of patients with a positive fecal occult test, this mechanism cannot be completely ruled out as a likely explanation for our results.

Moreover, a study on the iron status in hypertensive patients indicates that groups receiving CCB treatment show lower ferritin levels [24].

A study by Zhang et al. showcases an additional explanation for the mechanism behind decreased iron levels. [25] It indicates that treatment with CCBs leads to a potentially significant reduction (40–100%) of high divalent metal transporter–1 (DMT–1) levels in the liver of iron-overloaded mice [26]. DMT–1 is an integral membrane protein responsible for the transportation of iron and other divalent metals through the cell membrane. DMT–1 is also present in the membrane on the apical surface of duodenal enterocytes and is involved in the absorption of inorganic iron at this level [27]. Further research is needed to demonstrate whether a decrease in DMT–1 expression in duodenal cells is present at the duodenal enterocyte level or whether this outcome is similar in organisms in the absence of iron overload.

As this study seems to be the first to indicate an association between CCBs and anemia risk in patients with heart failure, the specific mechanism behind these findings is yet to be determined. Possible explanations include EPO resistance determined by blocking the cellular calcium influx [22], gastrointestinal bleeding [23], and/or impaired iron absorption through intestinal cell by reduced DMT–1expression [25].

### 4.3. Angiotensin-Converting Enzyme Inhibitor and Iron Metabolism

Additionally, we detected a weak correlation between ACEI treatment and low hematocrit levels (*p* = 0.04).

Similar findings have been found in various other studies. A meta-analysis of seven studies covering 29,061 patients has shown a significant association between anemia and ACEI use, with an overall 1.56-fold (95% CI, 1.40–1.73, I_2_ = 17%) increased anemia risk [28]. There are a limited number of mechanisms accounting for this association. Firstly, it is known that angiotensin II stimulates erythroid precursors. The use of ACEI decreases circulating angiotensin II levels, thus inhibiting the erythroid precursors [29]. Secondly, other studies have demonstrated that ACEI lower insulin growth factor levels, an action associated with erythroid stimulation [30]. Finally, the levels of the erythropoiesis inhibitor *N*-acetyl-seryl-aspartyl-lysine-proline rise with ACEI use, causing a fall in erythropoiesis and anemia [31].

### 4.4. Limitations of the Study

One limitation to our study was the small number of patients included in the cohort, as the essential inclusion criteria were the diagnosis of iron deficiency and heart failure. Patients with other causes of anemia were excluded, thus limiting the number of subjects included in the study. We could not confidently exclude some confounding factors, such as nutritional status, that might have influenced the present findings. However, the results seem not to be influenced by the heart failure severity.

Another limitation is the lack of follow up. We were able to collect data from the patients at admissions in our clinic, but the difficulties of long-term monitoring made follow up impossible.

These results are in agreement with similar studies [9,12,17,27] and will hopefully stimulate further clinical trials aimed at clarifying the suggested hypothesis, mainly that different types of drugs used for patients with heart failure can contribute to iron deficiency.

## 5. Conclusions

Several recent studies have raised questions regarding the influence of different drugs on iron metabolism, and even more questions with respect to the mechanism involved. The contribution of our research is defined by the additional data regarding a probable drug-induced etiology of iron deficiency. Practitioners should therefore be aware of the potential impact of therapeutic recommendations, as this may imply more intensive monitoring of the biochemical parameters of iron deficiency in such patients.

## Figures and Tables

**Table 1:** Clinical, biological and echocardiographic group profile depending on beta blockers use.

Parameter	Patients Using Beta Blockers *n* = 87		Patients Not Using Beta Blockers *n* = 41		*p* value
	Mean ± SD	Median IQR 25–75	Mean ± SD	Median IQR 25–75	
**Age**	72.78 ± 11.56	76(66–81)	73.32 ± 9.3	72(67–79.5)	0.996
**Hemoglobin (g/dL)**	12.37 ± 1.62	12.3(11.6–13.6)	12.61 ± 4.62	11.75(10.9–13.1)	0.142
**Hematocrit (%)**	38.67 ± 4.63	39.2(35.7–41.5)	38.61 ± 8.52	36.9(35.2–41.3)	0.120
**MCV (fl)**	86.69 ± 6.25	88.1(82–91.3)	84.84 ± 12.68	87.7(81.37–91.75)	0.992
**Iron (µg/dL)**	38.26 ± 12.1	38(29–46)	38.5 ± 12.1	40.5(29–47)	0.787
**Ferritin (ng/mL)**	87.99 ± 58.52	76(39–126)	83.22 ± 152.71	49(31–87)	0.028 *
**NT-proBNP (pg/mL)**	5232.32 ± 6482.89	2413(764–8270)	3537.27 ± 7106.92	1326(285–2791)	0.024 *
**CRP (mg/dL)**	2.7 ± 5.78	1.06(0.46–2.43)	1.8 ± 4.79	0.57(0.22–1.11)	0.425
**Creatinine (mg/dL)**	1.02 ± 0.37	0.93(0.76–1.18)	0.93 ± 0.04	0.86(0.72–1.03)	0.268
**Creatinine clearance (CKD-EPI)**	70.05 ± 23.28	70(54–84)	73.94 ± 20.5	69(59.5–87.75)	0.442
**LVEDD (mm)**	53.55 ± 8.73	52(47.5–60)	54 ± 10.37	53.5(46.5–62.25)	0.8
**LVESD (mm)**	40.57 ± 9.74	40(33–46)	38.6 ± 10.48	36.5(31.25–44)	0.322
**LVEF (%)**	45.06 ± 10.43	45.5(38–52)	49.79 ± 13.47	50(40–58.5)	0.057
**IVS (mm)**	11.28 ± 0.2	11(10–12.75)	11.96 ± 0.28	12(11–13)	0.131
**Posterior wall (mm)**	11.16 ± 1.74	11(10–12)	11.54 ± 1.47	12(11–12.87)	0.139
**NYHA Class**	2.61 ± 0.578	3(2–3)	2.46 ± 0.552	2(2–3)	0.143

CRP—C-reactive protein; IVS—interventricular septum; IQR—interquartile range; LVEF—left ventricular ejection fraction; LVEDD—left ventricular end diastolic diameter; LVESD—left ventricular end systolic diameter; MCV—mean corpuscular volume; NT-proBNP—N-terminal pro-brain natriuretic peptide; NYHA—New York Heart Association; SD—standard deviation; * *p* < 0.05.

**Table 2:** Correlation between the drugs investigated and anemia biological parameters.

Parameter	CCB		ACEI		Beta Blockers	
	r	*p* Value	r	*p* Value	r	*p* Value
**Hemoglobin**	–0.16	0.07	–0.16	0.07	0.005	0.67
**Hematocrit**	–0.23	0.008 *	–0.017	0.04 *	–0.03	0.95
**Iron**	–0.22	0.012 *	0.05	0.57	–0.009	0.91
**Ferritin**	–0.04	0.6	–0.10	0.22	0.023	0.8

CCB—calcium-channels blockers; ACEI—angiotensin-converting enzyme inhibitors; r—correlation coefficient; * *p* < 0.05.

**Table 3:** Clinical, biological, and echocardiographic group profiles depending on amlodipine use.

Parameters	Patients Using Amlodipine *n* = 36		Patients Not Using Amlodipine *n* = 92		*p* Value
	Mean ± SD	Median IQR 25–75	Mean ± SD	Median IQR 25–75	
**Age**	71.03 ± 11.86	75.5(62.3–80.8)	73.71 ± 10.41	76(67–81)	0.328
**Hemoglobin (g/dL)**	11.69 ± 1.86	11.8(10.6–12.6)	12.74 ± 3.17	12.4(11.6–13.67)	0.018 *
**Hematocrit (%)**	36.34 ± 5.5	36.9(33.9–38.3)	39.54 ± 6.07	39.8(36.5–41.7)	0.003 *
**MCV (fL)**	84.06 ± 5.47	83.8(80.4–88.5)	86.89 ± 9.67	88.9(83.52–91.8)	0.002 *
**Iron (µg/dL)**	34.02 ± 11.24	35(28–44)	39.97 ± 1.25	40(30.25–48.75)	0.038 *
**Ferritin (ng/mL)**	79.07 ± 52.12	70.8(31–120.75)	89.26 ± 109.4	70(38–100)	0.939
**NT-proBNP (pg/mL)**	2252.15 ± 3264.42	829(390–2379)	5673.28 ± 7391.87	2689(710–6801)	0.008 *
**CRP (mg/dL)**	2.25 ± 3.03	0.8(0.34–3.88)	2,49 ± 6.26	0.99(0.34–1.87)	0.914
**Creatinine (mg/dL)**	1.00 ± 0.38	0.9(0.75–1.17)	0.99 ± 0.33	0.89(0.75–1.09)	0.994
**Creatinine clearance (CKD-EPI)**	69.62 ± 23.67	70(51–85)	71.9 ± 22.05	69.5(57.25–88)	0.473
**LVEDD (mm)**	51.06 ± 7.04	51(45–55)	54.72 ± 9.84	54(48–60)	0.069
**LVESD (mm)**	37.03 ± 8.52	35(28.75–44.25)	41.18 ± 10.34	40(34–46.5)	0.066
**LVEF (%)**	49.89 ± 10.64	50.5(40.75–56)	45.39 ± 11.82	46.5(37–52.5)	0.108
**IVS (mm)**	11.37 ± 1.71	11(10–13)	11.56 ± 1.73	12(10.25–12.87)	0.694
**Posterior wall (mm)**	11.81 ± 1.71	12(10.37–13)	11.06 ± 1.6	11(10–12)	0.057
**NYHA Class**	2.44 ± 0.607	2(2–3)	2.61 ± 0.554	3(2–3)	0.157

CRP—C-reactive protein; IVS—interventricular septum; IQR—interquartile range; LVEF—left ventricular ejection fraction; LVEDD—left ventricular end diastolic diameter; LVESD—left ventricular end systolic diameter; MCV—mean corpuscular volume; NT-proBNP—N-terminal pro-brain natriuretic peptide; NYHA—New York Heart Association; SD—standard deviation; * *p* < 0.05.

**Table 4:** Clinical, biological, and echocardiographic group profile depending on ACEI use.

Parameters	Patients Using ACEI *n* = 48		Patients Not Using ACEI *n* = 80		*p* Value
	Mean ± SD	Median IQR 25–75	Mean ± SD	Median IQR 25–75	
**Age**	73.25 ± 10.51	76(67–79.75)	72.46 ± 11.52	73(66–82)	0.681
**Hemoglobin (g/dL)**	12.09 ± 1.4	11.9(11.2–13.2)	13.05 ± 4.33	12.4(11.52–13.87)	0.097
**Hematocrit (%)**	37.82 ± 4.13	37.8(35.1–40.7)	40.05 ± 8.24	39.8(35.7–42.9)	0.063
**MCV (fL)**	86.62 ± 7.07	88.1(82.1–91.6)	85.27 ± 11.09	85.75(81.67–91.1)	0.528
**Iron (µg/dL)**	38.81 ± 11.74	40(31–46)	37.56 ± 12.63	38(28–47)	0.589
**Ferritin (ng/mL)**	78.42 ± 57.66	66(31–100)	100.11 ± 140.51	74(44–119)	0.333
**NT-proBNP (pg/mL)**	3759.18 ± 5470.21	1773(498.25–4660.5)	6264.73 ± 8086.14	2324.5(681.75–10597.5)	0.203
**CRP (mg/dL)**	2.61 ± 6.13	0.97(0.23–2.25)	2.1 ± 4.33	1.03(0.54–1.96)	0.447
**Creatinine (mg/dL)**	0.99 ± 0.36	0.85(0.73–1.17)	1 ± 0.32	0.93(0.79–1.07)	0.447
**Creatinine clearance (CKD-EPI)**	71.4 ± 22.91	70(56–88)	71.07 ± 21.87	68.5(57.25–85.75)	0.883
**LVEDD (mm)**	52.81 ± 7.99	52(47.5–58)	55.21 ± 11.04	54(47–62.25)	0.392
**LVESD (mm)**	38.08 ± 8.15	37.5(32–44)	43.29 ± 12.04	39(34–54)	0.081
**LVEF (%)**	48.28 ± 9.53	49.5(40–55.25)	43.77 ± 14.16	44.5(31.75–54.25)	0.086
**IVS (mm)**	11.44 ± 1.68	11(10.12–13)	11.62 ± 1.81	12(10–12.5)	0.824
**Posterior wall (mm)**	11.33 ± 1.63	11(10–12)	11.2 ± 1.72	11(10–12.5)	0.761
**NYHA Class**	2.48 ± 0.551	2(2–3)	2.71 ± 0.582	3(2–3)	0.053

ACE—angiotensin-converting enzyme inhibitors; CRP—C-reactive protein; IVS—interventricular septum; IQR—interquartile range; LVEF—left ventricular ejection fraction; LVEDD—left ventricular end diastolic diameter; LVESD—left ventricular end systolic diameter; MCV—mean corpuscular volume; NT-proBNP—N-terminal pro-brain natriuretic peptide; NYHA—New York Heart Association; SD—standard deviation.
